# Guaiacol Externally as Are Antipyretic

**Published:** 1896-11

**Authors:** 


### GUAIACOL EXTERNALLY AS AN ANTIPYRETIC.

Dr. Horace G. McCormick, in the June number of the *Therapeutic Gazette*, recapitulates his experience of the past two years in the local use of this drug. The applications (864 in all) were made on forty-three different persons, of both sexes and various ages. The largest dose was twenty-five drops; the smallest, two drops. In typhoid fever it is certain to reduce the temperature and slow and strengthen the pulse and respiration. With ordinary care, he says, it is absolutely safe and non-injurious, being much more convenient and acceptable than the cold bath, while fully as effective. The patient sweats considerably, but will have no chills unless the temperature is reduced below 100°.

The application is made in the right iliac region, which should first be thoroughly cleansed and dried. The guaiacol is then dropped slowly on the surface and rubbed in with the hand for from ten to fifteen minutes, after which the part may be covered with oiled silk or waxed paper. Local irritation is produced but rarely; if it occurs, the site of the application must be changed. The effect of inunction is shown within thirty minutes, and lasts from three to four hours. It is a curious fact that guaiacol internally, while perhaps the best of intestinal antiseptics, does not reduce the temperature nearly as much as when locally applied. The administration of the drug in medicinal doses by either method does not affect the normal pulse and respiration. For internal use the carbonate of guaiacol in 2½ grain doses dry on the tongue is preferable. The local applications should be employed as needed in conjunction with internal administration.

